# Towards Conceptual Clarification of Proactive Inhibitory Control: A Review

**DOI:** 10.3390/brainsci12121638

**Published:** 2022-11-29

**Authors:** Wery P. M. van den Wildenberg, K. Richard Ridderinkhof, Scott A. Wylie

**Affiliations:** 1 Department of Psychology, University of Amsterdam, Nieuwe Achtergracht 129 B, 1018 WS Amsterdam, The Netherlands; 2 Amsterdam Brain and Cognition (ABC), University of Amsterdam, Nieuwe Achtergracht 129 B, P.O. Box 15900, 1001 NK Amsterdam, The Netherlands; 3 Department of Neurosurgery, University of Louisville, Louisville, KY 40202, USA

**Keywords:** proactive inhibition, reactive inhibition, inhibitory control, motor inhibition

## Abstract

The aim of this selective review paper is to clarify potential confusion when referring to the term proactive inhibitory control. Illustrated by a concise overview of the literature, we propose defining reactive inhibition as the mechanism underlying stopping an action. On a stop trial, the stop signal initiates the stopping process that races against the ongoing action-related process that is triggered by the go signal. Whichever processes finishes first determines the behavioral outcome of the race. That is, stopping is either successful or unsuccessful in that trial. Conversely, we propose using the term proactive inhibition to explicitly indicate preparatory processes engaged to bias the outcome of the race between stopping and going. More specifically, these proactive processes include either pre-amping the reactive inhibition system (biasing the efficiency of the stopping process) or presetting the action system (biasing the efficiency of the go process). We believe that this distinction helps meaningful comparisons between various outcome measures of proactive inhibitory control that are reported in the literature and extends to experimental research paradigms other than the stop task.

## 1. Introduction

This paper presents a brief and selective review of the literature on proactive inhibitory control over behavior. It mainly seeks conceptual clarification and is by no means meant to be exhaustive. A PubMed literature search in July of 2022 for the keywords “proactive inhibit*” in the publication title or the abstract yielded an outcome of 570 results, about 25% of which have been published in the last five years, underscoring a rising interest in the topic. One potential source of confusion with this topic is that sometimes the term proactive inhibitory control refers to various mechanisms and other times to a performance outcome (i.e., the success or failure of an action being inhibited). Often, these various distinctions are used interchangeably, which led to our consideration of a rather simple framework that might offer conceptual clarity and consistency in studies of proactive inhibitory control. The framework is introduced within the context of the response-stopping paradigm (i.e., the stop-signal task), although we later discuss later how the framework may be useful in response conflict paradigms (e.g., Simon task, Flanker task) as well.

Let us first define proactive inhibitory control as adaptive preparatory processes that modulate or control the chances of inhibition success in the near future. In this sense, the proactive nature refers to processes mobilized in advance of upcoming situations in which the need for inhibition may be called upon. Moreover, this definition adopts the position that proactive inhibitory control refers to the outcome or success of inhibition, irrespective of the specific mechanism or process that may be mobilized to achieve the outcome. Most usages of proactive inhibitory control in the literature, including our prior work, seem to conflate mechanism and outcome. However, as will become clear from a brief inventory of definitions that have been used in the literature, these adaptive inhibitory control processes are multifaceted and may refer to different underlying mechanisms, all of which impact the success or failure outcome of inhibitory control. Defining proactive inhibitory control in terms of inhibition success, irrespective of the mechanism at play, does not imply that we are not interested in the underlying mechanisms. Quite to the contrary, we hope to clarify the different guises that proactive inhibition can take by focusing on these mechanisms and processes. Focusing on the outcome allows us to consider proactive inhibition under one unified umbrella definition, while focusing on underlying processes allows us to clarify its different uses in the literature, a clarification that we deem useful if not urgent.

Before we elaborate on the framework of proactive inhibitory control, we first focus on the concept of reactive inhibitory control, which provides the mechanism by which an action is ultimately cancelled or stopped. Thus, this paper will start with presenting a brief overview of behavioral correlates of reactive inhibitory control, as measured by the renowned stop-signal paradigm and its variants. The second part integrates the concept of proactive inhibitory control as a set of processes that bias the likelihood that reactive inhibitory control will be successful in any situation in the immediate future. For the sake of conceptual clarification, we distinguish between the following two general classes of proactive control processes that shape the success of inhibiting an action: pre-amping reactive inhibition versus pre-setting action. Pre-amping inhibition refers to the adaptive process of amplifying (i.e., up or down) the reactive inhibition mechanism to increase or decrease, respectively, the proficiency of stopping an action should the need to do so present itself in the immediate future. The second manifestation, pre-setting action, refers to the adaptive tuning of action-related processes other than reactive inhibition per se to decrease or increase the difficulty of stopping an action should the need to do so present itself in the imminent future. Note that both mechanisms modulate the outcome of the reactive inhibition process by biasing its chances for success or failure. We will argue that for assessing the manifestations of proactive inhibitory control in previous and future studies, it will be useful to consider this taxonomy because of its consequences for interpretation and implications.

In the following, we present a brief inventory of annotations in the literature to reactive inhibitory control, as measured by the stop-signal paradigm. Next, serving conceptual clarification, we present a selective overview to distinguish between two manifestations of proactive inhibitory control, namely pre-amping reactive inhibition versus pre-setting the action system. A concluding section reiterates the advantages of this conceptual interpretation when comparing results across studies on response inhibition, including experimental paradigms other than the stop-signal task.

## 2. Reactive Inhibition

**Definition 1** **(Reactive Inhibition).** *Reactive inhibition is the adaptive mechanism that stops ongoing motor actions abruptly or on the fly. Reactive inhibition can be triggered by external events, such as a stop signal, or by changed internal goals, such as the voluntary decision to stop.*

### 2.1. Reactive Inhibition as Immediate Stopping Control over Actions

Imagine that you are going to get a coffee from the cafeteria in the psychology department where you have been working for the past 24 years. During these 24 years, you have walked that same route from your desk to the coffee corner countless times so that you could even find your way blindfolded. However, upon turning a corner you suddenly halt and come to a full stop while stumbling upon a red sign hanging on the hallway door, indicating “Passage Temporarily Closed Due to Construction”. This example illustrates the need for the ability to inhibit or stop ongoing behavior if the situation calls for it [1,2]. Throughout this review paper, we will refer to this mechanism of impromptu, on-the-fly stopping control over actions as reactive inhibition. Note that the term “reactive inhibition” in its original form has been used to indicate inhibition that results from executing some other process [1]. Examples are inhibition of return [3] and negative priming, where inhibition is triggered as a consequence or side-effect of concurrent processing. It is reactive because it is a direct reaction to the processing of exogenous environmental cues, such as a “do not enter” sign on the door in the example above, or to goal changes generated endogenously, such as arresting your action to open the door of the local grocery story because you suddenly realize that you are not wearing a required face mask. In sports, reactive inhibition is the mechanism that allows hitters to abruptly stop or cancel their initiated swing upon judging that the ball will not cross the strike zone or allows soccer players to suddenly stop their initial movement to take a shot and pass instead. Currently, the term reactive inhibition is used primarily to indicate stopping, arresting, or interrupting ongoing actions as a means of adaptive control over behavior, often as part of action override (suppressing one course of action in favor of another). This description of reactive inhibition will be used throughout this review. Ultimately, reactive inhibition is the mechanism that determines whether an action is stopped or not.

### 2.2. The Stop-Signal Task Measures Reactive Inhibition

**Figure 1:**
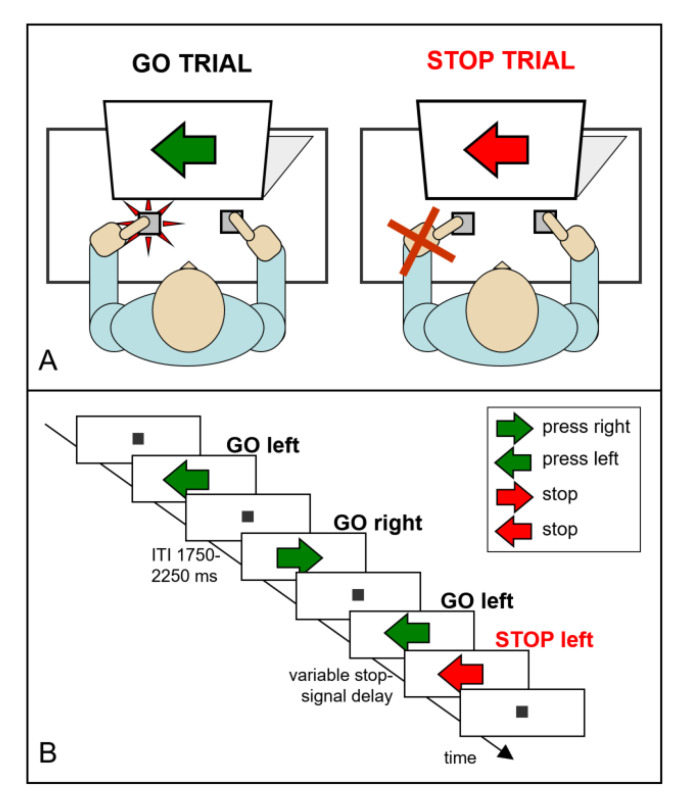
Stop-task design. (**A**) Participants were instructed to press the left or right button in the direction indicated by the green arrow (i.e., go trials). (**B**) On 30% of the trials, the color of the arrow changed from green to red (i.e., stop trials) upon which participants should inhibit their go response.

The foremost and most well-established research paradigm to study reactive inhibitory control over actions is the stop-signal paradigm [1,2,4,5]; for pioneering work, see [6,7]. For an extensive literature review of research using the stop-signal task, the reader is referred to overview papers by Verbruggen and colleagues [8,9]. In short, the standard version of the stop task requires participants to discriminate between two visual go signals that are serially presented in (semi-) random order (see Figure 1). For example, the instruction might be to quickly press a left response button with the left index finger whenever an X is presented on a computer screen and to press a right response button with the right index finger when the letter O is presented. In addition to these go task requirements, the need to inhibit is introduced by the instruction to inhibit or stop the button-pressing response upon the infrequent and unpredictable presentation of the stop signal, such as a brief auditory tone or a color change of the green go signal to red, which may be presented shortly after the onset of the go signal.

Performance on stop-signal trials has been conceptualized as a horse race between two processes, the go and stop processes, which are triggered by the onsets of the go signal and the stop signal, respectively [2]. If the go process wins the race, inhibition fails and the overt response is executed. However, if the stopping process wins the race, the motor response is successfully stopped. The nature of the horse race has been the subject of investigation. The independent race model conceptualizes the race between going and stopping with stochastic finishing times. Conversely, interactive model versions have been proposed in which go and stop processes act as stochastic accumulators that interact with each other [10,11,12]. Yet, other models have proposed a negative stochastic dependence between go and stop processes [13]. For the interested reader, Schall and colleagues have provided a succinct review of stop-task models [12].

**Figure 2:**
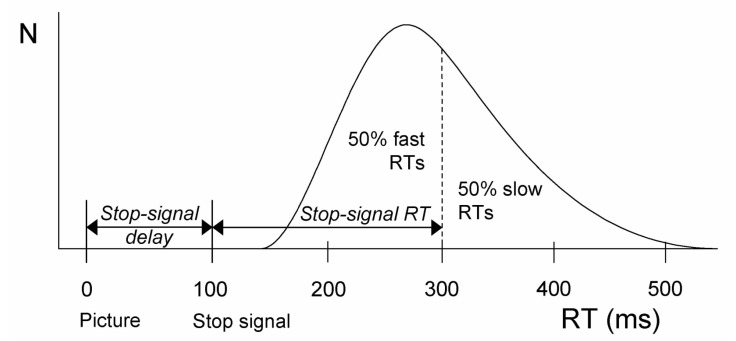
Integration method. Calculation of stop-signal RT (SSRT) according to the race model (Logan and Cowan, 1984). The black curve depicts the distribution of RTs on go trials (i.e., trials without a stop signal), representing the finishing times of the go process. Assuming independence of the go and stop processes, the finishing time of the stop process bisects the go RT distribution. Here, responses could not be stopped on 50% of the stop trials. Hence, the n-th go RT that represents the finishing time of the stop process is 300 ms. Go RTs shorter than 300 ms will win the race (resulting in failed stop trials), whereas go RTs longer than 300 ms will lose the race against the stopping process (resulting in successful stop trials). Here, subtracting mean stop-signal delay (100 ms) from the 50th percentile of go RT (300 ms) yields an estimated SSRT of 200 ms.

A major advantage of the stop-signal task over other experimental paradigms that tap into inhibitory control, such as the Go/Nogo and Stroop tasks, as well as more complex neuropsychological tests, such as the Wisconsin Card Sorting Task, is that the latency of the covert response-inhibition process, the stop-signal reaction time (SSRT), can be estimated within the conceptual and computational framework of the race model (see Figure 2) and that there is wide consensus on standards for experimental control and reporting [9]. Again, the computational approaches to estimating SSRT are varied, including nonparametric (e.g., [14]), as well as Bayesian parametric methods (e.g., [15,16,17]). As mentioned above, the behavioral dependent measure of reactive inhibition per se is stop-signal reaction time, or SSRT. For healthy young adults, SSRT typically ranges from 175–250 ms. Significantly slower stopping latencies have been reported for children and elderly participants [18]. Stopping latencies are also prolonged in clinical populations, such as children diagnosed with attention-deficit hyperactivity disorder (ADHD) [19], diagnosed with OCD [20], and in children expressing primary complex motor stereotypes, such as involuntary, complex, repetitive, and apparently purposeless movements [21]. Clinical adult populations showing prolonged stopping include those diagnosed with Parkinson’s disease [22,23]. The prolonged use of substances, such as cannabis or cocaine, can also impair the latency of reactive inhibition [24,25].

In sum, SSRT derived from the stop-signal paradigm has proven to be very useful for quantifying individual differences in the efficiency of reactive stopping control. Next, we turn to the conceptual framework related to proactive inhibition as the preparatory processes modulating the success or failure of reactive inhibition.

## 3. Proactive Inhibition: Pre-Amping Reactive Inhibition

**Definition 2** **(Proactive inhibition as pre-amping reactive inhibition).** *Proactive inhibition can be expressed by “amping up” the reactive inhibition system to increase the chances of successful stopping in the near future.*

### 3.1. Amping Up Reactive Inhibition

Suppose you are in a hurry to be on time for an important appointment. Traffic is growing increasingly busy and your anxiety is elevating. Just ahead, the traffic light turns orange, and you instinctively punch the gas to make it through the intersection before it turns red. Confidence in your quick wits gives way to panic and doubt as the flash of a traffic-camera in your rear-view mirror catches your attention—did the camera catch you? With no time to stop and think, the next traffic-light is already fast approaching and might turn orange at any moment. Harboring concerns about tickets and fines, one strategic option is to maintain your speed (you are still in rush, after all), but heighten your readiness to stop pressing the gas pedal so you can quickly switch to the brake pedal if the light suddenly turns orange.

This scenario illustrates the need for proactive inhibition, where amping up your readiness to inhibit without altering any go- or action-related processes increases the speed and likelihood of successful inhibition. The purpose of this proactive adaptation strategy is to facilitate the efficiency of reactive inhibitory control in case of an upcoming, anticipated stop signal (i.e., the onset of an orange traffic light in the example above). Referring to the race model underlying cognitive processing in the stop-signal paradigm, this strategy of preparatory proactive inhibition in essence biases the race in favor of stopping efficiency. The intended net effect will be an increase in the probability and speed of successful reactive inhibition when the situation calls for it. Studies that report ‘proactive inhibition’ typically refer specifically to the preparatory amping up of reactive inhibition processes (e.g., [26,27]).

Pre-amping reactive inhibition is typically studied using variations in the standard stop-signal task as well. In such cases, it is measured as the degree to which SSRT is affected by the experienced likelihood of an upcoming stop signal. A straightforward test of expectation and preparatory effects on the efficiency of reactive stopping is provided by comparing the latency of reactive inhibition (SSRT) derived from a condition with relatively low stop-signal probability (e.g., 17% of the trials) versus a condition in which stop-signal probability is relatively high (e.g., 33% of the trials) [28,29]. For example, Federico and Mirabella varied stop-signal probability according to the (left or right) movement side. Results indicate that participants were faster to withhold movements toward the side where stop signals were more frequent [30]. In general, participants stop faster in the higher stop-probability condition, compared to the lower stop-probability condition. This decrease in reactive stopping latency in relation to changes in stop-signal probability signifies the ability to proactively amp up the efficiency of the reactive inhibition process as a function of the context in order to increase reactive stopping success.

Another example is provided by Zandbelt and colleagues [27] who used a variant of the Slater–Hammel paradigm [31], termed the stop-signal anticipation task (see also [32,33]). This modified version has the advantage that the presentation of the go signal can be separated in time from the presentation of the cue indicating stop-signal probability. This enabled the dissociating of cue-related processing (i.e., the effect of stop-signal probability), on the one hand, and stop-signal processing, on the other hand. Participants pressed a response button with their right thumb in order to stop a moving bar as close to the fixed target location as possible. They were instructed to stop (i.e., withhold pressing the button) if the moving bar halted before reaching the target. Before the start of each trial, a cue was presented that informed the participant about the probability of a stop signal on that trial. Symbols indicated a stop-signal probability on that trial of either 0%, 24%, or 35%. Interestingly, the three stop-signal probability conditions yielded similar SSRTs. Thus, although participants were informed about the likelihood of stopping, they did not amp up their inhibition process to the extent that SSRTs were shorter in the context with higher stop-signal probability. Note that Zandbelt and colleagues manipulated stop-signal probability on a trial-by-trial basis (that is within a block of trials), which might have increased the demands on preparation processes due to frequent variations. Alternatively, the comparison of between-block variations in stop-signal probability did result in a proactive amping up of reactive inhibitory control (as illustrated by Castro-Meneses and colleagues [28]). Another design-related difference worth noting is that participants performing the stop-signal anticipation task executed a timed response, rather than a speeded response as in the standard stop-signal task.

Chikazoe and colleagues however provided a clear case of the proficient amping up of reactive stopping [34]. They employed a standard stop task and added color cues to go signals. A yellow cue indicated a traditional “uncertain-go” trial, during which participants were instructed to respond by pressing a button with the thumb while being prepared to inhibit if a stop signal is presented. Additionally, the other half of the go signals were blue, indicating a “certain go” trial without a stop signal. Their analyses suggest that participants who used the cue information (those who prepared) had shorter SSRTs than those participants who did not. The conclusion was that preparation before the presentation of the stop signal contributed to the improved efficiency of stopping.

Alternatively, in several other paradigmatic examples of the pre-amping of inhibition, the level of reactive inhibition may be said to be premeditated, in that the resolve to not act in a certain way under certain conditions was made ahead of time. Such examples include the cold pressor test (involving a premeditated and sustained resolve to resist the strong impulse to remove one’s hand from the ice-cold water [35]; delay discounting, involving a premeditated and sustained resolve to forego immediate gratification in favor of a larger future reward [36]; and deception, involving a premeditated determination to not reveal veridical information because of the prioritization of some other interest [37].

The ability to amp up reactive inhibition was evident from a series of stop-signal experiments performed by Bissett and Logan, who observed that stopping latency was shorter on stop trials that immediately followed another stop trial [38]. Interestingly, this post-signal improvement of reactive inhibition turned out to be modality specific. That is, SSRT improved only if the two subsequent stop signals were presented within the same modality (e.g., sequential presentation of two auditory stop trials) but was absent if stop-signal modality changed (e.g., an auditory stop tone following a visual stop signal). This amping up of reactive inhibition after having encountered a stop trial within the same modality could either be the result of strategic adjustments, such as a shift toward the stopping goal, or be explained in terms of more automatic processing as in stimulus repetition effects, often referred to as priming (for a discussion, see [38]). Note that amping up reactive inhibition after stop trials must be compensated by amping down after no-stop trials. In comparison with amping up after stop trials, amping down after no-stop trials may be less steep because there are usually (many) more no-stop trials than stop trials, thus serving something akin to a gradual return to baseline.

Pre-amping reactive inhibition seems at play when participants receive a monetary reward to perform well on the stop task. SSRT is generally shorter when participants are motivated [39]. Interestingly, reward also lowered the incidence of trigger failures of the inhibition process [40], contributing to more efficient stop-signal processing with rewards.

In a large study involving over 12,000 participants, ranging in age between 18 and 60+, Smittenaar and colleagues confirmed that the latency of reactive inhibition (SSRT) to stop signals increased from about 365 ms for the group of 18–24-year-olds to about 411 ms for the 60+ group [41]. This provides a clear replication of the age-related decline in reactive stopping control reported by Williams and colleagues [18]. In addition, Smittenaar et al. noted that males showed a more pronounced deterioration of the ability to stop as age increased, compared to females. Importantly, presenting visual cues that informed participants whether the left or the right thumb press might be stopped in response to a stop signal afforded proactive control. For comparison, stopping latencies to cued stop trials varied from 340 ms for young adults to 365 ms for the oldest age group. Here, proactive control was quantified as the difference between reactive inhibition (SSRT to unprepared stop trials without cue) and reactive inhibition derived from cued stop trials (SSRT to prepared stop trials). Importantly, cue-related preparation shortened the latency of upcoming reactive inhibition, and this was interpreted as a clear indication of effective proactive inhibitory control. Interestingly, and opposed to the pattern observed for reactive inhibition, proactive inhibitory control defined as the benefit of amping up reactive stopping did not vary over the course of healthy aging. Overall, females showed better proactive control, compared to males, irrespective of age [41].

As mentioned above, neurodegenerative conditions, such as Parkinson’s disease, impair inhibitory control [22]. However, the reactive inhibition deficit during early stages of Parkinson’s disease can be ameliorated by pharmacological therapy such as levodopa intake [42,43] or surgical interventions such as in deep-brain stimulation of the subthalamic nucleus [44,45,46,47,48]. Another example of a clinical study assessing pre-amping inhibition is provided by Atkinson-Clement and colleagues [49]. They administered the stop-signal task to myoclonus dystonia patients with and without deep-brain stimulation of the globus pallidus interna (compared to healthy controls). Pre-amping inhibition, assessed through the influence of several consecutive go or stop trials on reactive inhibition success, was shown to be impaired in unoperated patients, but this impairment appeared remedied in patients with deep-brain stimulators.

### 3.2. Amping Down Reactive Inhibition

When discussing proactive control, we typically consider increases in control, such as amping up reactive inhibition, as described in the previous section. However, the dimension of proactive control may also include ‘letting go’, ‘letting one’s guard down’, or in general adopting more lenient levels of control. Whether this leniency takes the form of active amping down reactive inhibition or a more passive lack of investment remains to be studied, but it is evident that what goes up must come down. As an illustration, consider the previous traffic example in which you hurry to make it to an important appointment in time. Alas, due to road works blocking the way, you have to take an alternative route that is unfamiliar to you. You are being redirected by a series of arrow signs that are planted along the roadside, but these are hardly visible because of the twilight hour. The passenger next to you, commenting incisively on the obvious fact that you risk being late, taxes your limits even more. You find yourself slamming the brakes just in time for a red traffic light, the presence of which has been brought to your attention by the squabbling passenger blurting out “STOP!”. Conceivably, under these demanding concurrent circumstances, your reactive inhibition control system might be amped down to the extent that you were slow to stop to the red traffic light itself and needed an additional auditory cue to trigger the reactive inhibition process.

This down-scaling of reactive inhibition has been illustrated by modifications of the classical stop-signal task in order to capture more subtle manifestations of reactive inhibition control, as opposed to the all-or-none form of stopping. Whereas the standard version requires a full stop of ongoing actions, the stop-change variant captures the ability to stop an ongoing action and initiate an alternative action instead upon the presentation of an external stimulus [4,50]. The additional requirement to execute an alternative response, as derived from the stop-change task, generally increases stopping latencies, compared to the SSRT obtained in the standard stop-signal task [51,52,53,54]. For a discussion of various strategies that underly cognitive processing in the stop-change task, see [55].

In addition, task variants drawing upon selective stopping capabilities may involve the selective inhibition of certain responses or selective inhibition to certain stimuli. An example of selective response stopping is the instruction to stop when hearing the stop tone but only if you are about to issue a button press response with the left hand. Conversely, a right-hand button press response should not be inhibited upon a stop signal and should always be completed [54,56,57]. SSRT derived from a selective stopping context (e.g., “try to stop your left-hand response”) is generally longer compared to all-or-none stopping latencies. This prolongation is observed when adding the requirement to stop selectively might be an indication of proactively amping down reactive inhibition in favor of the response-selection process to determine if either the right or the left hand has to be initiated. Alternatively, an instance of selective stimulus stopping is introduced by the requirement to discriminate between a valid stop signal (for example a high-pitched tone of 1000 Hz) versus an invalid signal of lower frequency (say 500 Hz). Go responses, therefore, should only be inhibited if followed by a high-pitched stop signal; the low tones can be completely ignored [58,59,60]. SSRT derived from selective stopping paradigms is typically prolonged compared to SSRT obtained from the standard stop task, indicating additional demands on inhibitory processing and the use of various strategies (see [61]).

Importantly, amping reactive inhibition up or down is often reported in relation to changes in the reactions to the go signal. For example, amping up reactive inhibition is often reported together with a strategic slowing of reactions to the go signal [8,61,62,63]. This brings us to the next potential manifestation of proactive inhibitory control, namely presetting action-related processes in order to increase the chances of successful reactive stopping control in preparation for the presentation of upcoming stop signals.

## 4. Proactive Inhibition: Pre-Setting the Action Control System

**Definition 3** **(Proactive inhibition as pre-setting the action system).** *Proactive inhibition can be expressed by an advanced presetting of the action system to increase the chances of successful reactive inhibition if needed in the future.*

### Presetting Action-Related Process to Facilitate Future Inhibition Success

Recall the real-life example from the previous section. You were pressed to be on time for your appointment and just accelerated to run an orange traffic light. You cut it close this time but do not want to risk a traffic penalty. Instead of maintaining speed and being extra prepared and vigilant to stop (i.e., proactively amping up reactive inhibition), this time you decide to slow down to ensure that you will be able to stop should the light turn orange; slowing down will also prevent the need to suddenly override your rushed action in the first place. Better safe than sorry.

We refer to this proactive control mechanism as pre-setting action-related processes (in this case, slowing down) to prepare to suppress your action, should the need arise in the immediate future, in order to increase reactive inhibition success. The purpose of this proactive strategy is (again) to facilitate the efficiency of reactive stopping control in case of an upcoming stop signal (i.e., the onset of an orange traffic light). Like pre-amping reactive inhibition, pre-setting action processes also biases the outcome of the race between going and stopping processes in favor of the stopping process. The intended net effect will be an increase in the probability of successful reactive inhibition if the situation calls for it. Note that the mechanism underlying this variant of proactive inhibitory control is not reactive inhibition per se. Instead, (p)re-setting action-related processes benefits the efficiency of reactive inhibition as an indirect or secondary effect. Importantly, action may also be pre-set in opposite directions. For instance, a riskier action strategy may make one less prepared for suppressing one’s action, should that need arise. In this case, pre-setting action-related processes biases the race between going and stopping processes in favor of the go process. The intended net effect will be a decrease in the probability of successful reactive inhibition should the situation call for it.

The research paradigm of choice for measuring reactive inhibition and the proactive amping up of reactive inhibition, the stop-signal paradigm, also allows the quantification of the proactive pre-setting of actions. In the stop task, the latter type of proactive control is often expressed as the slowing down of responses to go signals, despite the explicit task instruction not to prolong go reaction time, in an effort to increase the chances of successful inhibition should the go signal be followed by a stop signal.

The proactive slowing of responses on go trials might be the direct result of increasing the frequency of a stop signal, compared to a task condition in which stop signals were presented less often [50,61,62,64]. Recall that modulation of stop-signal frequency has also been associated with amping up or amping down reactive inhibition [28,29]. In addition, responses on go trials are typically prolonged if the go trial was immediately preceded by a stop trial, compared to when the go trial was preceded by another go trial [8,63]. This post-stop-signal slowing of responses was observed irrespective of inhibition success related to the preceding stop trial, thereby following both successful stopping and unsuccessful stopping. Bissett and Logan replicated these findings and pointed to the involvement of memory-related processes by showing that the post-stop-signal slowing of go RT was more pronounced if the same go signal was presented on two consecutive trials (i.e., go signal repetition), compared to situations in which the go signals alternated [61].

Another typical quantification of presetting action control as a proactive strategy to increase stopping success involves the comparison of performance in two type of tasks. Participants complete a standard version of the stop-signal task in which choice trials are typically randomly mixed with stop trials. In addition to SSRT as an index of the efficiency of reactive stopping control, the stop task also provides mean reaction latencies to the go signal (i.e., go RT), reflecting the efficiency of action-related processes. Importantly, participants also complete a pure choice RT task that is similar in design, compared to the stop-signal task, with the exception that stop signals never occur in the pure choice task and, therefore, never require reactive inhibition. Proactive control is typically indexed by the relative lengthening of go RT in a stop-signal context, compared to go RT in a pure choice task, a context without stop trials [63,64]. A different approach worth highlighting has been developed by Mirabella and colleagues using a setup that requires participants to move their arms towards a target [65]. Instead of indexing proactive inhibition merely as RT lengthening, they measured proactive inhibition by comparing RT and movement times (MT) on go trials in a stop task with similar movements in a simple RT task without stop signals. This approach is based on the reciprocal relationship between the two behavioral parameters RT and MT. Interestingly, compared to performance on the simple-RT task, go RT in the stop task is prolonged, whereas MT is significantly shorter. This phenomenon, dubbed “the context effect” represents an optimization of motor strategy in the two different contexts and has been explained as follows [65]. In the stop task, the anticipated presentation of a stop signal induces RT lengthening on go trials, which benefits the coding of movement parameters (i.e., MT). Conversely, the short RTs on go trials in a simple RT context do not profit from this additional time benefit. Here, the movement plan unfolds during the execution of the motor response, causing a lengthening of MT. It is this lengthening that indexes proactive inhibitory control. The main advantage of this approach with respect to other designs assessing proactive inhibition, such as the conditional stop signal [66], the stop-signal anticipation tasks [67], or the or the classic Chikazoe’s design [34] is a reduced load on attentional and working memory. Apparently, some of the experimental manipulations already discussed in Section 3.1 about the proactive amping up of reactive inhibition also have an effect on the latency of go responses. For example, increasing the frequency of stop signals within a block of trials slows down responses to go trials. Along the same lines, presenting participants with cues associated with a relatively high imminent probability of a stop signal not only results in the proactive amping up of reactive inhibition (as in shorter SSRT [34]) but also increases response latencies to go signals that were preceded by such a cue but were not followed by a stop signal. The response latency on these so-called “uncertain” go trials were prolonged, compared to responses to certain go trials of which the participant knew that these were never followed by a stop signal. This proactive slowing of go responses as a function of stop-signal probability context was also reported by Zandbelt and colleagues [27]. Using the stop-signal anticipation task, they observed the shortest go response latencies on go trials that were cued with 0% stop-signal probability, with incremental slowing as cues conveyed contexts of 24% and 35% stop-signal probability.

## 5. Concluding Remarks

We have described reactive inhibition as the mechanism by which action is stopped and differentiated proactive mechanisms that bias the likelihood of reactive inhibition success. However, it is worth considering that proactive mechanisms are always at play in situations where a reaction might need to be inhibited. That is, there is a context to every situation that affords preparatory processes, amped up or down, primed or slackened, perhaps even shut off at times, that place reactive inhibition in a better or worse place for success. Considering the standard stop-signal task that generally recommends a context where 25% of trials deliver a stop signal, changing the frequency of stop signals alters reaction and stopping speeds. Thus, proactive inhibitory control reflects the state of preparatory processes that influence the ultimate success or failure of inhibiting a reaction. If the context is held constant, one is better able to quantify reactive inhibition efficiency with minimal preparatory adjustments (apart from trial-by-trial adjustments). If the context is varied, one has to consider the potential engagement of pre-amping and/or presetting processes. Even within a consistent context (for example, 25% stop signals), there is room for proactive adjustments on a trial-by-trial basis, even though the macro proactive strategy might be fairly constant.

Using the basic framework presented here can help clarify the language, design, and inferences of future studies interested in studying the role of proactive processes in modulating the success of response inhibition. At a minimum, studies of reactive inhibition must carefully record and acknowledge the state and biases of preparatory processes given the context. When studies attempt to modulate proactive control mechanisms, it is helpful to specify whether variables are pre-amping the reactive control mechanism or pre-setting action-related processes.

The distinction between pre-amping reactive inhibition and pre-setting action may perhaps seem almost trivial. However, given the state of the literature in which proactive inhibition is treated as an undifferentiated unitary construct while actually pooling together different processes under the same head, we think such a conceptual distinction helps to disentangle these processes, disambiguate what we mean when we talk about proactive inhibition, and clarify our view on this consequential topic. This approach could help determine whether experimental conditions (e.g., speed/accuracy balance instruction, reward/punishment, working memory load, peer pressure, etc.), interventions (e.g., alcohol consumption, working memory training, mindfulness programs, medication regimes, etc.), or individual/group differences (e.g., addiction, education level, impulse control disorder, treatment categories, etc.) affect the ability to pre-amp reactive inhibition, pre-set action, or both. Interestingly, the selective impairment of reactive versus proactive inhibitory control can mark the phenotype of distinct clinical conditions or even different phases of disease [68]. For example, children diagnosed with ADHD are associated with a specific impairment in reactive but not in proactive motor inhibition [67], a finding that has recently been replicated by Suarez and colleagues [69]. A comparable pattern has been reported by Mirabella et al. [21] in children expressing primary motor stereotypes, showing a deficit in reactive motor inhibition, compared to typically developing children, whereas proactive control seems to be intact. Mancini et al. [20] showed that whereas children diagnosed with OCD have an impairment in both reactive and proactive motor inhibition, Tourette’s patients expressing tics have near the expected level of motor inhibition. Alternatively, autism spectrum disorder (ASD) without comorbidities has been associated with intact reactive inhibition, together with a specific deficit in proactive control strategies [70]. Of particular interest is the observation that Parkinson’s disease affects the two domains of inhibitory motor control differently as the disease progresses. Early-stage PD (i.e., Hoehn and Yahr stage 1) has been associated with impaired reactive stopping, leaving proactive inhibition relatively intact [71]. Notably, the progression of the disease to Hoehn and Yahr stage 2 marks the onset of a deficit in proactive inhibitory control [72]. These relevant clinical patterns demonstrate that reactive and proactive inhibitory control are meaningful operational concepts that can be used to mark pathophysiological phenotypes and disease progression. Note that while the paradigmatic focus of the current framework has been on the stop-signal task, the framework may also be valuable to conceptual approaches in response conflict tasks, where a strong impulse to react must be suppressed in favor of a more appropriate goal-directed response. One might engage proactive inhibition to selectively amp up the suppression mechanism to improve the proficiency of thwarting an incorrect response impulse, or one might pre-set action by slowing down reaction speed or modulating focused attention to the goal-driven stimulus–action features in order to reduce the chances of reacting impulsively to an irrelevant stimulus–action process. Examples of pre-amping suppression or pre-setting action in conflict tasks have been demonstrated in studies of frequency effects [73], speed-accuracy tradeoffs [74,75], with clinical modulation from medications [76,77], and deep-brain stimulation [78].

At this point, it is worth mentioning the term pre-emptive inhibition [79]. Pre-emptive inhibition indicates pre-empting the need to engage reactive inhibitory control processes in the future to increase the chances of the selection of goal-directed actions. In our preparation of this framework, we wrestled with the idea of pre-emptive mechanisms that might be deployed to eliminate the need for reactive inhibition. Pre-empting inhibition in the stop-task is discouraged by the typical instruction to participants to maintain a consistently speeded response strategy to go signals, that is, not to slow down in anticipation of the possible presentation of a stop signal. Nevertheless, a Bayesian processing approach revealed manifestations of pre-emptive inhibition on stop trials referred to as trigger failures [80]. These deficiencies in triggering the inhibition process upon the presentation of a stop signal seem more common in clinical groups, such as in children diagnosed with ADHD [81] or when task processing demands are high.

Proactive inhibition, whether through pre-amping reactive inhibition or pre-setting action, typically affects the probability of successfully suppressing an action impulse. Conceptually, however, proactive inhibition might also apply to the probability of successfully suppressing ongoing processes other than action. For instance, proactive inhibition might serve to suppress one’s fears in bungee-jumping. Proactive inhibition might even serve to suppress action inhibition, for instance in cliff-diving, where one may proactively suppress the tendency to stop running at the last second before reaching the edge; or in baseball hitting, where one may proactively decide to go after a pitch and suppress the tendency to inhibit a swing, even when the pitch is wide. These alternative targets of proactive inhibition may again be accomplished through either pre-amping reactive inhibition or pre-setting action.

In sum, different researchers mean different things when talking about proactive inhibition. When approaching a traffic light that might turn orange, one strategy is to maintain your speed while preparing to stop pressing the gas pedal (and slam the brakes instead) if the light suddenly turns orange; another is to slow down to ensure that you will be able to stop should the light turn orange. Although both strategies are considered as expressions of proactive inhibition and both aim to bias the outcome towards increased probability of stopping if the light turns orange, their underlying mechanisms are quite different. One involves pre-amping reactive inhibition; the other involves pre-setting the parameters of our actions and reactions. Both can save lives, but via different approaches. By exploring these roads, we hope to have clarified what it is we talk about when studying proactive inhibition.

## Data Availability

Not applicable.

## Abbreviations

ADHD	attention deficit/hyperactivity disorder
ms	milliseconds
MT	movement time
OCD	obsessive compulsive disorder
PD	Parkinson’s disease
RT	reaction time
SSRT	stop-signal reaction time
